# Syndemic factors associated with non-fatal overdose among young opioid users in New York City

**DOI:** 10.3389/fpubh.2023.1195657

**Published:** 2023-06-26

**Authors:** Honoria Guarino, David Frank, Kelly Quinn, Dongah Kim, Krista Gile, Kelly Ruggles, Samuel R. Friedman, Pedro Mateu-Gelabert

**Affiliations:** ^1^Institute for Implementation Science in Population Health, CUNY Graduate School of Public Health & Health Policy, New York, NY, United States; ^2^Behavioral Science Training Program in Drug Abuse Research, NYU Rory Myers College of Nursing, New York University, New York, NY, United States; ^3^Department of Population Health, New York University School of Medicine, New York, NY, United States; ^4^Department of Mathematics and Statistics, University of Massachusetts Amherst, Amherst, MA, United States

**Keywords:** overdose, opioid use, injection drug use, hepatitis C virus (HCV), syndemic, young adults, polysubstance use

## Abstract

**Introduction:**

Rates of illicit opioid use are particularly high among young adults, yet research on overdose experience and factors associated with overdose in this population remains limited. This study examines the experiences and correlates of non-fatal overdose among young adults using illicit opioids in New York City (NYC).

**Methods:**

539 participants were recruited via Respondent-Driven Sampling in 2014-2016. Eligibility criteria included: aged 18–29 years old; current residence in NYC; and nonmedical prescription opioid (PO) use and/or heroin use in the past 30 days. Participants completed structured interviews to assess their socio-demographics, drug use trajectories, current substance use and lifetime and most recent overdose experiences, and were tested on-site for hepatitis C virus (HCV) antibodies.

**Results:**

43.9% of participants reported lifetime overdose experience; of these, 58.8% had experienced two or more overdose events. The majority of participants’ most recent overdoses (63.5%) were due to polysubstance use. In bivariable analyses, after RDS adjustment, having ever overdosed was correlated with: household income of >$100,00 growing up (vs. $51,000-100,000); lifetime homelessness; HCV antibody-positive status; lifetime engagement in regular nonmedical benzodiazepine use, regular heroin injection and regular PO injection; and using a non-sterile syringe in the past 12 months. Multivariable logistic regression identified childhood household income >$100,00 (AOR=1.88), HCV-positive status (AOR=2.64), benzodiazepine use (AOR=2.15), PO injection (AOR=1.96) and non-sterile syringe use (AOR=1.70) as significant independent correlates of lifetime overdose. A multivariable model with multiple overdoses (vs. one) found only lifetime regular heroin use and PO injection to be strong correlates.

**Discussion:**

Results indicate a high prevalence of lifetime and repeated overdose among opioid-using young adults in NYC, highlighting a need for intensified overdose prevention efforts for this population. The strong associations of HCV and indices of polydrug use with overdose suggest that prevention efforts should address the complex risk environment in which overdose occurs, attending to the overlapping nature of disease-related risk behavior and overdose risk behavior among young people who inject opioids. Overdose prevention efforts tailored for this group may find it useful to adopt a syndemic conception of overdose that understands such events as resulting from multiple, and often interrelated, risk factors.

## 1. Introduction

Opioid-involved overdoses have increased dramatically in the U.S. in the last 20 years and continue to be a major public health crisis (1, 2). From 1999 to 2010, prescription opioid (PO)-involved overdose deaths quadrupled (3), paralleling major increases in medical prescribing of opioids for chronic pain and associated diversion and nonmedical use of POs (4). In the past decade, heroin use more than doubled among young adults ages 18–25, and, in 2015, heroin was the most common opioid involved in opioid-related overdose deaths nationwide (1, 4). Since 2014, there has been a sharp increase in overdose deaths associated with illicitly manufactured fentanyl, a potent opioid that is now commonly present as an adulterant in heroin and other illicit drugs (1, 5). In New York City (NYC), the rate of unintentional drug overdose deaths increased by 143% from 2010 to 2016 (6). In 2016, heroin was the most common substance involved in overdose deaths, present in 55% of decedents; fentanyl was involved in 44% of overdose deaths, and opioid analgesics other than fentanyl were involved in 18% (6).

Young people under age 30 are a key population of interest in this overdose crisis. These youth came of age when diverted POs were widely available; as a result, many were introduced to opioid use through the nonmedical use of POs (7–9). One study of heroin users in treatment found that 75% of those who began using opioids after 2000 initiated use with POs as compared to those who began using opioids in the 1960s when more than 80% initiated opioid use with heroin (10). As policies and practices intended to curb PO misuse, such as Prescription Drug Monitoring Programs (PDMPs), abuse-deterrent PO formulations and increased law enforcement, made POs more difficult and expensive to acquire, many nonmedical PO users transitioned to heroin use and, more recently, the use of fentanyl (7, 11–14).

Young opioid users may be at particularly high risk for overdose for several reasons. First, previous studies have shown that young adult PO users often use in social situations such as parties where polydrug use—a behavior linked to increased risk of overdose—is common (9, 14–16). Second, while young opioid users usually begin taking opioids orally or intra-nasally (sniffing, snorting), they often transition to injection drug use, a route of administration associated with increased risk of overdose (15, 17, 18). Finally, many young adult opioid users may not utilize harm reduction services such as syringe exchange programs (SEPs), and thus may be less informed on effective strategies to prevent and/or respond to an overdose event such as the use of naloxone than older users (19, 20).

Despite the salience of opioid overdose as a national public health concern, there has been limited research examining the overdose experiences and risk factors of the new generation of young opioid users. Existing studies have identified demographic factors associated with increased risk of overdose among illicit opioid users such as being White (21, 22), childhood poverty and homelessness (9, 19, 22–24), chronic hepatitis C virus (HCV) infection (21), as well as behaviors including drug injection (9, 22, 25), polydrug use (21, 26), recent stimulant or tranquilizer use (9, 24), and injection equipment-sharing (23). However, overdose research among young adult opioid users often focuses on specific sub-groups such as people who inject drugs (22, 25), heroin users (27) or homeless and high-risk drug users (9), and may not describe the specific overdose risks of young adult opioid users who do not meet these criteria.

The young adult participants in the present study represent a diversity of socio-economic backgrounds, as well as drug use and injection behavior. Although participants did not all initiate opioid use with POs, they are members of a distinct, age-based cohort that began their drug-using careers while PO use and overdose rates were rapidly increasing—a factor which shaped their drug use patterns, views, and trajectories. This is particularly important since previous research has found that young opioid users are often outside of older heroin-using networks and may see themselves and their drug use activity as different from that of more traditional heroin users (11, 13, 14). The present study is also distinctive because the participants comprise a community sample who were not recruited through their affiliation with a drug treatment or harm reduction program.

The goals of this paper are: (1) to assess the prevalence of non-fatal overdose in an urban, community-recruited group of young adults who are currently using illicit opioids; and (2) to determine significant sociodemographic and drug-related correlates of non-fatal overdose in this group. Focusing on non-fatal overdose is of critical importance, as prior research has shown that recent experience of non-fatal overdose is one of the most robust predictors of a future fatal overdose (28–30).

## 2. Materials and methods

### 2.1. Recruitment procedures

This study collected survey data from 539 young adult (ages 18–29) opioid users living in NYC in 2014–2016. Participants were recruited via Respondent-Driven Sampling (RDS), a form of chain-referral sampling designed to connect with difficult-to-reach populations that utilizes participants’ network connections to drive recruitment (31, 32). We initially recruited 20 eligible young adult opioid users as RDS “seeds.” Seeds were recruited by multiple means; some were participants in the study’s formative qualitative phase, while others were identified through street-based recruitment or via referrals from local service providers and other research studies. After completing a screening and the structured interview, seeds were asked to refer up to three eligible peers from their networks. This peer-referral process was repeated with the seeds’ recruits and for successive sample waves thereafter. For this analysis, the 20 seeds are included within the sample of 539.

Eligible participants met the following criteria: nonmedical use of POs and/or heroin use in the past 30 days; current residence in a borough of NYC; aged 18–29 years old; English-speaking; and ability to provide informed consent. Opioid use was confirmed with a multi-modal screening protocol that included a verbal questionnaire, a point-of-care urine test for opiates, oxycodone and methadone, a visual quiz to identify pictures of common PO pills, and, for participants who reported injecting drugs, visual inspection for injection marks. Potential participants who appeared to be 25 or older were asked to show photo identification to confirm age. Eligible participants provided written informed consent. Participants were compensated $60.00 for completing the interview and received an additional incentive for each eligible peer they referred to the study. At the start of recruitment, participants were paid $20 for each referral; later, to increase the number of referrals per participant, incentives were increased to $40, $50, and $60 for first, second and third referral, respectively. All procedures were approved by the Institutional Review Board of National Development and Research Institutes, Inc.

### 2.2. Data collection

Participants completed a computer-assisted, interviewer-administered survey, lasting 90–120 min. The survey instrument included socio-demographic and behavioral questions, including items regarding participants’ drug use histories, lifetime overdose experiences, past 12-month and past-30-day substance use and, where applicable, injection practices. A detailed set of items queried participants about the circumstances and sequelae of their most recent overdose event. Participants’ HCV status was assessed with point-of-care rapid antibody testing. Small samples of participants’ blood were obtained by finger-prick and tested with the OraQuick Advance Rapid HCV Antibody Test (manufactured by OraSure Technologies, Inc., Bethlehem, PA).

### 2.3. Variables

Any lifetime overdose was a binary variable from the self-reported response to a question about whether participants had ever “lost consciousness, stopped breathing, or was unresponsive as a result of taking prescription and/or non-prescription drugs by any route of administration.” Events involving non-opioid substances were also included.

Sociodemographic variables included: age; race/ethnicity; household income growing up; education; and lifetime homelessness. HCV status was also included.

Seven binary drug use variables were examined. Regular use was defined as three or more times a week for at least 1 month, and drug binging was defined as “took considerably more of any drug(s) than usual or mixed more drugs than usual in one sitting.” Lifetime variables included regular nonmedical use of benzodiazepines, POs and heroin, and regular injection of heroin and POs (“PO injection” refers to a process in which opioid pills intended for oral consumption, including “abuse-deterrent” formulations intended to resist such manipulation, are broken down through persistent crushing and mixing with water into a sufficiently liquid form that can be injected with a syringe). Additional variables were receptive use of a non-sterile syringe in the past 12 months and drug binging in the past 30 days.

We present univariable descriptives for sociodemographic variables with all response choices (Table 1), as well as several additional variables characterizing participants’ opioid use trajectories and overdose experiences (Tables 1, 2).

**Table 1:** Sociodemographic and opioid use characteristics of young adult opioid users in New York City, 2014–16, *N* = 539.

	Sample prevalence Number (percent)	Population estimate Percent (standard error)
**Sociodemographics**		
Age mean (standard deviation) Gender (*n* = 530, 9 participants missing data)	24.0 (3.1)	–
Male	356 (67.7%)	69.1% (±4.2)
Female	170 (31.5%)	30.1% (±4.2)
Transgender	4 (0.8%)	0.8% (±0.3)
**Race** ^a^		
White/Caucasian	371 (68.8%)	67.0% (±5.1)
Black/African American	42 (7.8%)	9.0% (±3.7)
Asian/Pacific Islander	7 (1.3%)	1.6% (±0.7)
Native American	9 (1.7%)	1.1% (±0.4)
Multiracial	43 (8.0%)	6.1% (±1.2)
Missing	67 (12.4%)	–
Hispanic/Latino	154 (28.7%)	28.8% (±4.97)
**Household income growing up (annual)**		
≤$50,000	227 (42.1%)	43.8% (±4.9)
$50,001–100,000	176 (32.7%)	34.0% (±4.7)
>$100,000	102 (18.9%)	17.4% (±3.0)
Do not know or missing	34 (6.3%)	–
**Education (*n* = 538, 1 participant missing data)**		
Did not complete high school	107 (19.9%)	23.1% (±4.7)
High school graduate or GED	224 (41.6%)	36.0% (±4.9)
Some college	181 (33.6%)	33.0% (±4.1)
College graduate or higher	26 (4.8%)	7.4% (±2.5)
Ever homeless	309 (57.4%)	55.2% (±4.9)
Hepatitis C antibody-positive	105 (19.6%)	19.1% (±3.9)
**Opioid use**		
**Lifetime opioid use characteristics**		
Heroin only (never used POs)	6 (1.1%)	2.7% (±3.4)
POs only (never used heroin)	89 (16.5%)	17.1% (±4.7)
Used heroin and POs	444 (82.4%)	80.2% (±5.3)
**Past-30-day opioid use (*n* = 528, 11 participants missing data)**		
Heroin only	195 (36.9%)	43.0% (±4.8)
POs only	108 (20.5%)	20.8% (±4.7)
Heroin and POs	225 (42.6%)	36.2% (±4.3)
**Opioid use trajectory (among the 444 who had used both POs and heroin, *n* = 438, 6 participants missing data)**		
Used heroin before POs	33 (7.5%)	6.8% (±2.7)
Used POs before heroin	338 (77.2%)	76.9% (±4.2)
Used heroin and POs at same age	67 (15.3%)	16.2% (±3.5)

POs, prescription opioids. Most of the missing values for Race are due to participants identifying their race, as well as their ethnicity, as Hispanic/Latino.

^a^Most of the missing values for Race are due to participants identifying their race, as well as their ethnicity, as Hispanic/Latino.

**Table 2:** Overdose characteristics of young adult opioid users in New York City, 2014–16, among those with lifetime experience of overdose (*N* = 234).

	Sample prevalence Number (percent)	Population estimate Percent (standard error)
Lifetime overdose among total sample (*n* = 539)	234 (43.4%)	46.6% (±14.6)
Age at first overdose Mean (standard deviation)	20.4 (3.4)	–
**Number of overdoses (*n* = 233, 1 participant missing data)**		
1	96 (41.2%)	38.9% (±11.8)
2–3	76 (32.6%)	39.6% (±11.5)
4 or more	61 (26.2%)	21.5% (±9.1)
**Number of substances used at last overdose (*n* = 233, 1 participant missing data)**		
1	85 (36.5%)	41.1% (±11.4)
2	85 (36.5%)	37.3% (±11.4)
3 or more	63 (27.0%)	21.6% (±10.5)
**Opioid(s) used at last overdose (*n* = 234)**		
Heroin only	162 (69.2%)	75.8% (±9.6)
PO only	28 (12.0%)	10.7% (±8.3)
Heroin and PO	19 (8.1%)	5.8% (±4.5)
No opioid^a^	25 (10.7%)	7.6% (±3.4)
**Route of heroin administration at last overdose (among the 181 who used heroin at last overdose, *n* = 178, 3 pts. missing data)**		
Injected	149 (83.7%)	80.8% (±11.8)
Snorted/sniffed	29 (16.3%)	19.2% (±11.8)
Smoked	0 (0%)	0% (±0)
**Route of PO administration at last overdose** ^**b**^		
Injected	5 (17.2%)	7.4% (±9.0)
Snorted/sniffed	5 (17.2%)	11.7% (±18.5)
Smoked	1 (3.5%)	9.1% (±29.6)
Oral	21 (72.4%)	75.8% (±29.8)

PO, prescription opioid.

a Per the study’s operational definition of “overdose,” events that involved only non-opioid substances (e.g., alcohol, benzodiazepines, stimulants, etc.) were included, if they involved loss of consciousness and/or respiration or the person was unresponsive.

b Percentages and population estimates total >100 because participants were able to report multiple routes.

### 2.4. Data analysis

Initial analyses were conducted in R versions 3.2.2 and 3.2.4 (R Core Team). We used the R package MICE (33) to impute missing network size data for a portion of the sample. Population estimates for key variables were then calculated with the successive sample estimator (34) in the R package RDS (35), using a working population size of 15,000. Results were not sensitive to working population size.

We estimated bivariable associations for potential correlates with lifetime overdose in two ways: (1) using SAS 9.4 (SAS Institute, Inc., Cary, NC), we estimated odds ratios (OR) and 95% confidence intervals (CI) with logistic regression models; and (2) using R, we calculated *p*-values for permutation tests of the correlations. Permutation tests were developed by study investigators (36) and are included as a measure of validity since in an RDS sample standard tests are subject to bias due to the dependent nature of selection. This test uses a dependent permutation distribution to estimate the expected distribution of a test statistic in a population with a sampling and dependence structure like the one observed, but with no association between the variables. This developing statistical technique does not yet allow for multivariable modeling; therefore, our models may produce biased estimates. We estimated adjusted odds ratios (AOR) with a logistic regression model that included all variables that were associated with the outcome at the level of *p* ≤ 0.05. We estimated ORs and AORs in a similar manner for an additional model that predicted multiple overdoses among the subsample with any lifetime overdose (*n* = 233).

## 3. Results

### 3.1. Descriptive characteristics

#### 3.1.1. Socio-demographics

Participants’ socio-demographic and selected drug use characteristics are presented in Table 1, along with RDS-adjusted estimates for the study’s target population of young adult opioid users in NYC. Participants were 67.7% male (population estimate [PE]: 69.1%), 68.8% (PE: 67%) white, and had a mean age of 24 years old. A notable minority (18.9%; PE: 17.4%) reported an annual household income of over $100,000 while they were growing up, and 38.4% (PE: 40.4%) had attended at least some college, yet 57.4% (PE: 55.2%) had been homeless at some point in their lifetime. Nearly one-fifth of the sample (19.6%; PE: 19.1%) tested HCV antibody-positive. Most participants (82.4%; PE: 80.2%) had used both heroin and POs in their lifetime; 16.5% (PE: 17.1%) were exclusive PO users who had never used heroin, and only 1.1% (PE: 2.7%) were exclusive heroin users who had never used POs. The majority of participants (77.2%; PE: 76.9%) initiated PO use before initiating heroin use, and 79.5% (PE: 79.2%) reported using heroin in the past 30 days. For most variables, RDS population estimates are within one-two percentage points of the sample frequencies, suggesting that the sample is fairly representative of young opioid users in NYC.

#### 3.1.2. Overdose experiences

Nearly half of participants (43.4%; PE: 46.6%) reported having ever experienced a non-fatal overdose. Of that sub-set, 58.8% (PE: 61.1%) had experienced two or more overdoses in their lifetime, and 26.2% (PE: 21.5%) had experienced 4 or more lifetime overdoses. These participants reported experiencing their first overdose at an early age (*M* = 20.4 years old; SD = 3.4).

The majority of participants reported polysubstance use immediately preceding their most recent overdose; 63.5% (PE: 58.9%) had used two or more substances at last overdose, while 27% (21.6%) had used three or more substances. The top three non-opioid substances used at participants’ last overdose were (in rank order): benzodiazepines; alcohol; and marijuana. With regard to opioids, participants’ most recent overdoses were primarily heroin-involved: 69.2% (PE: 75.8%) reported heroin as the only opioid used prior to their most recent overdose, and 8.1% (PE: 5.8%) reported using both heroin and POs at their most recent overdose. Heroin use at last overdose was also closely linked to the injection route of administration. Among participants whose most recent overdose involved heroin, 83.7% (PE: 80.8%) reported having injected the drug, while only 17.2% (PE: 7.4%) of those whose last overdose involved POs reported having injected POs at that time. Participants’ overdose characteristics and associated RDS-adjusted population estimates are presented in Table 2. Again, discrepancies between the sample frequencies and the population estimates are generally small (within 5 percentage points, on average), supporting the representativeness of the sample.

#### 3.1.3. Overdose outcomes and perceived causes (results not in tables)

Among those who had experienced overdose, 61.5% (*n* = 144) reported an average of 2.6 emergency department (ED) visits (range 1–23, SD = 3.5). Forty-two percent reported an average of 2.1 hospital admissions (range 1–17, SD = 2.3). Reported reasons for the most recent overdose included mixing too many drugs (*n* = 62), using more than their usual dose (*n* = 56), and the heroin being stronger than usual/expected (*n* = 44).

### 3.2. Bivariable associations with overdose

Bivariable and multivariable relationships of characteristics and behaviors with lifetime non-fatal overdose are presented in Table 3. Standard odds ratios indicate that white non-Hispanic participants (vs. Hispanics), those who grew up in households earning over $100,000/year (vs. $50,001–100,000/year), and those who had ever been homeless had significantly higher odds of lifetime overdose. Some education had a protective effect, such that participants who had graduated high school or obtained a GED had lower odds of overdose than those with lower educational attainment. Notably, HCV antibody-positive status was associated with more than five times the odds of overdose relative to HCV antibody-negative status.

**Table 3:** Bivariable and multivariable associations of sociodemographic and drug-related correlates and any lifetime overdose (*n* = 533)^a^.

	Overdose prevalence	Bivariable associations			Multivariable associations^d^	
Correlates	Number (%)	OR (95% CI)	OR *p*-value	Permutation test for correlate *p*-value	AOR (95% CI)	AOR *p*-value
Total	234 (43.9)	NA	NA	NA	NA	NA
**Age**						
18–23	96 (40.0)	ref	ref	ref	NA	NA
24–29	138 (47.1)	1.34 (0.95, 1.89)	0.10	0.29	NA	NA
**Gender** ^b^						
Male	152 (42.2)	ref	ref	ref	NA	NA
Female	80 (47.3)	1.23 (0.85, 1.78)	0.27	0.17	NA	NA
**Race/ethnicity**						
White non-Hispanic/Latino	174 (53.1)	2.89 (1.91, 4.37)	<0.0001	0.10	1.94 (1.15, 3.29)	0.01
Non-white, non-Hispanic/Latino	17 (32.7)	1.24 (0.63, 2.45)	0.53	0.46	1.51 (0.70, 3.26)	0.29
Hispanic/Latino	43 (28.1)	ref	ref	ref	ref	ref
**Household income growing up (annual)**						
≤$50,000	83 (36.7)	0.85 (0.56, 1.27)	0.42	0.30	1.21 (0.74, 1.97)	0.46
$50,001–100,000	70 (40.7)	ref	ref	ref	ref	ref
>$100,000	59 (58.4)	2.05 (1.24, 3.37)	0.005	<0.01	1.88 (1.06, 3.33)	0.03
Do not know/missing	22 (64.7)	2.67 (1.24, 5.75)	0.01	0.049	1.78 (0.70, 4.49)	0.22
**Education** ^b^						
Less than high school	55 (51.9)	ref	ref	ref	ref	ref
High school graduate or GED	84 (37.7)	0.56 (0.35, 0.89)	0.02	0.06	0.54 (0.31, 0.94)	0.04
Beyond high school	94 (46.3)	0.80 (0.50, 1.28)	0.35	0.32	0.55 (0.31, 0.97)	0.001
**Homelessness (lifetime)** ^b^						
Ever	165 (53.9)	2.72 (1.89, 3.91)	<0.0001	0.02	1.29 (0.82, 2.04)	0.27
Never	68 (30.1)	ref	ref	ref	ref	ref
**HCV antibody status**						
HCV+	79 (75.2)	5.29 (3.26, 8.60)	<0.0001	<0.01	2.64 (1.47, 4.74)	0.0004
HCV−	155 (36.5)	ref	ref	ref	ref	ref
**Regular nonmedical benzodiazepine use (lifetime)** ^b^^,^^c^						
Yes	159 (54.6)	2.68 (1.88, 3.84)	<0.0001	<0.01	2.15 (1.41, 3.30)	0.0004
No	74 (30.8)	ref	ref	ref	ref	ref
**Regular nonmedical prescription opioid use (lifetime)** ^c^						
Yes	201 (43.7)	0.94 (0.57, 1.55)	0.81	0.90	NA	NA
No	33 (44.2)	ref	ref	ref	NA	NA
**Regular heroin use (lifetime)** ^c^						
Yes	217 (50.7)	5.32 (3.06, 9.25)	<0.0001	0.24	1.23 (0.58, 2.64)	0.59
No	17 (16.2)	ref	ref	ref	ref	ref
**Regular heroin injection (lifetime)** ^b^^,^^c^						
Yes	181 (58.8)	4.69 (3.19, 6.88)	<0.0001	<0.01	1.52 (0.88, 2.62)	0.14
No	52 (23.3)	ref	ref	ref	ref	ref
**Regular prescription opioid injection (lifetime)** ^c^						
Yes	70 (76.1)	5.37 (3.21, 9.01)	<0.0001	<0.01	1.96 (1.07, 3.60)	0.03
No	164 (37.2)	ref	ref	ref	ref	ref
**Non-sterile syringe use (past 12 months)**						
Yes	92 (69.2)	4.08 (2.68, 6.21)	<0.0001	<0.01	1.70 (1.03, 2.81)	0.04
No	142 (35.5)	ref	ref	ref	ref	ref
**Drug binging (past 30 days)**						
Yes	178 (48.6)	1.87 (1.28, 2.75)	0.001	0.06	1.35 (0.86, 2.13)	0.20
No	56 (33.5)	ref	ref	ref	ref	ref

OR, odds ratio; AOR, adjusted odds ratio; CI, confidence interval; ref, referent; NA, not applicable.

a Six participants were missing data on overdose and were omitted from analyses.

b Total *n*’s for these variables are 1 or 2 participants <234 (the number of participants who reported lifetime overdose) due to missing data.

c “Regular” is defined as 3 or more times per week for at least 1 month.

d Multivariable model adjusted for all variables associated with overdose at *p* < 0.05 in bivariable models, including: race/ethnicity, income, education, homelessness, HCV, regular benzodiazepine use, regular heroin use, regular heroin injection, regular prescription opioid injection, non-sterile syringe use, and drug binging.

With regard to drug use behavior, participants who reported lifetime engagement in regular nonmedical benzodiazepine use, regular heroin use, regular heroin injection and regular PO injection had significantly increased odds of lifetime overdose relative to participants who had not regularly engaged in these forms of drug use. Overdose was also associated with use of a non-sterile syringe in the past 12 months and binging on drugs in the past 30 days.

For many of these correlates, the results of permutation tests to adjust for bias introduced by RDS recruitment confirmed the results of conventional odds ratio tests. However, in several cases, the significance level of a bivariable association was substantively affected, suggesting that the apparent association may be explained by the dependence in the sampling process. Specifically, white non-Hispanic race/ethnicity, high school/GED education level, regular heroin use and past-30-day drug binging were no longer significantly associated with overdose (at *p* < 0.05) after adjustment for RDS-related dependence.

### 3.3. Multivariable associations with overdose

In multivariable analysis, growing up in a high-income household (>$100,00/year) was associated with an 88% increase in odds of lifetime overdose relative to growing up in a middle-income household ($50,001–100,000/year). Among drug-related variables, regular nonmedical benzodiazepine use (AOR = 2.15; 95% CI: 1.41, 3.30), regular PO injection (AOR = 1.96; 1.07, 3.60) and non-sterile syringe use (AOR = 1.70; 1.03, 2.81) were associated with roughly two times the odds of overdose. HCV-positive status was identified as the strongest independent correlate of overdose, with participants who tested HCV-positive having more than two and one-half times the odds of lifetime overdose as those testing HCV-negative (AOR = 2.64; 1.47, 4.74). Although standard multivariable logistic regression indicated that white non-Hispanic race/ethnicity and high school/GED or higher educational attainment were significant correlates of overdose, permutation test results suggest that these associations are an artifact of RDS-induced sampling bias.

An additional multivariable model (results not shown in tables) identified correlates of multiple lifetime overdose (vs. one; subsample *n* = 233). Only regular heroin use and PO injection were significantly associated with multiple overdose in bivariable models. The multivariable model including both of these predictors indicated they were strongly associated with multiple overdose: regular heroin use OR = 4.03 (CI: 2.29, 7.06) and PO injection OR = 4.09 (2.42, 6.92).

## 4. Discussion

These findings demonstrate a high prevalence of non-fatal overdose and associated risk behaviors among opioid-using young adults in NYC. Drug-related behaviors including PO injection, nonmedical benzodiazepine use, and syringe-sharing were independently associated with lifetime overdose, as was HCV antibody-positive status.

The population estimate for lifetime prevalence of non-fatal overdose in our study, 46.6%, is significantly higher than rates reported among similar populations of young opioid users which generally range between 20 and 35% (9, 18, 19, 22). In part, this may reflect the unabated increases in opioid-involved overdose that have bedeviled the U.S. over the past 10-plus years (4). Silva et al.’s (9) and Liebling et al.’s (18) findings of lower rates of overdose may also reflect the specific nature of their samples which, respectively, included only exclusive PO users (not heroin users), or required participants to have recently used POs. Since heroin is unregulated, users can never know its potency or purity, thereby increasing risk of overdose (37). This risk has been dramatically compounded in recent years with the increasing presence of illicitly manufactured fentanyl as an adulterant in heroin and other drugs (38). However, fentanyl was a late arrival to the NYC drug scene relative to many other locations in the Eastern U.S. with less established heroin markets. Because fentanyl was involved in only a fairly small minority of overdose deaths in 2014 and 2015 (6), when the majority of this study’s data were collected, it was likely not a major driver of participants’ lifetime overdoses. Additionally, transition to heroin use is correlated with transition to injection drug use (13, 17), a route of administration that has been associated with increased risk of overdose (15)—a finding our bivariable results confirm.

Most participants who experienced overdose reported multiple overdose events, and about a quarter of those who had ever overdosed reported having experienced 4 or more overdose events. This finding is supported by previous research demonstrating that having previously overdosed is one the strongest predictors of a future overdose event (28, 39). Of particular note is the finding that participants experienced their first overdose at a young average age—about 20.

The majority of the most recent overdose experiences reported by participants were due to polysubstance use rather than use of only one drug. By far, heroin was the opioid most likely to be reported at last overdose. Among non-opioid substances used at last overdose, participants most frequently reported using benzodiazepines, alcohol and/or marijuana in combination with heroin and/or POs. As with some previous research, our study did not find an association between regular nonmedical PO use and non-fatal overdose (23, 40). However, there was an independent association of regular PO injection with overdose, a finding supported by recent studies in both rural and urban locations (9, 41, 42). These findings suggest that a comprehensive model of overdose risk that takes polysubstance use and route of drug administration into account may be best equipped to inform about and prevent overdose events.

PO injection appears to be functioning somewhat differently in the present group of young people who use/inject opioids than has been observed among older groups of opioid users in other areas of the U.S. In contrast to rural locations such as Kentucky (43, 44) and Scott County, Indiana (45) without established heroin markets, where PO injection is a fairly widespread, normative practice among nonmedical opioid users who inject, in this group of NYC young adults, PO injection is relatively uncommon (e.g., only 16% of participants reported lifetime *regular* PO injection; results not shown).

The ethnic/racial diversity of the study sample (nearly 30% Hispanic/Latino) and the lack of an association between race/ethnicity and overdose, after accounting for RDS-related sampling bias, may reflect recent trends both in NYC and nationally that indicate increasing involvement of young people of color in opioid misuse and its negative sequelae (6, 46). The observed association of overdose with having grown up in a household making over $100,000/year is a novel finding and possibly suggests that young opioid users from higher SES backgrounds may be at increased risk of overdose as a result of having greater access to disposable income with which to buy drugs.

The finding that HCV-infected participants had greater odds of lifetime overdose than uninfected participants can likely be understood as a function of the associations between overdose and injection drug use (and particularly regular PO injection) and use of non-sterile syringes—behaviors that present risk for HCV transmission (47). While we do not posit a causal relationship between HCV and overdose, HCV-positive status appears to be serving as a marker for a subgroup of young opioid injectors who are at particularly elevated risk for overdose and also engage in injection practices such as syringe-sharing and PO injection that present risk for HCV transmission (14). The emergence of lifetime regular benzodiazepine use as an independent correlate of overdose is consistent with existing research demonstrating that concurrent use of benzodiazepines and opioids markedly increases the risk of overdose (48, 49) and that polydrug use predicts overdose (24).

These results should be understood in light of some important limitations. Data for all variables except HCV antibody status were obtained by self-report and are thus subject to social desirability and recall bias. Further, because overdose, as well as many of the drug-related variables in our analysis are lifetime measures, their temporal inter-relationships cannot be determined. While some of the identified correlates may have a causal relationship with overdose, others may be markers for a subgroup of young opioid users who engage in a cluster of risky behaviors. Additionally, although RDS estimation procedures are intended to produce unbiased prevalence estimates for a given population, they are subject to some limitations (50) and present findings may not be generalizable to groups of young opioid users in other locations, given the specifics of the NYC setting. While the permutation tests used to assess bivariable associations were designed to account for the dependence in respondent-driven samples and in many cases confirmed the results of standard tests of association, some caution is nonetheless warranted in interpreting the results of the conventional odds ratio tests. Similarly, because the methods for multivariable analysis have not yet been established for RDS data, we used conventional logistic regression and acknowledge this as a limitation. In particular, some relationships may appear significant due to the dependence in the sampling structure.

### 4.1. Implications of study findings

A number of practical applications are suggested by these findings. The high rate of overdose speaks to the importance of expanding and mainstreaming overdose awareness and prevention efforts to general populations of youth. The high prevalence of repeated overdose points to the need to provide young people with overdose prevention and response education and naloxone in multiple settings, not only when they present for medical treatment following an overdose (e.g., in the ED), but also during routine contacts with primary care and other service providers.

The salience of polysubstance use in this population suggests that prevention efforts that focus on a single substance or drug class may not adequately address the complex risk environment in which overdose occurs. Rather, the clustering of overdose correlates, such as nonmedical benzodiazepine use, PO injection and non-sterile syringe use, suggests a synergistic relationship among multiple behavioral risk factors for overdose, as well as considerable overlap and possible synergy between risk factors for overdose and risk factors for transmission of blood-borne disease. Therefore, researchers and service providers may find it useful to adopt a syndemic understanding of opioid misuse, overdose, HCV/HIV and related health conditions, as has recently been suggested (51). A syndemic framework highlights the interactions among co-occurring diseases, as well as the social and environmental factors that produce vulnerability to them and/or exacerbate their consequences (52, 53).

A syndemic model that accounts for multiple, inter-related and multi-level risk factors could help to resist reductive, and often morally-based, drug scares that typically focus on a single behavior or disease at the expense of a nuanced understanding of the contexts in which drug use and drug-related behaviors occur. This may also open up discursive spaces to examine the harms experienced by people who use drugs (including overdose) as related to socio-structural factors, such as the availability of sterile syringes, law enforcement practices, policy measures and vulnerability due to homelessness, poverty and other consequences of social dislocation and disadvantage.

Adopting a multi-factorial model may also facilitate new forms of public health surveillance and intervention, particularly in regard to HCV. For example, given the strong correlation between overdose and HCV -positive status found in this study, as well as the fact that surveillance of acute HCV cases is lacking in much of the U.S., overdose fatality or morbidity data could potentially be used as an indicator to identify geographical areas where outbreaks of new HCV infections among young injectors might occur, thereby providing early warnings of potential HCV outbreaks to treatment providers and health care workers. Similarly, widespread efforts to saturate communities-at-risk with naloxone should be accompanied by parallel efforts to provide harm reduction education and sterile injection equipment to prevent HCV/HIV transmission, along with expanded access to low-threshold, non-punitive Medication-Assisted Treatment in those same localities.

## Data availability statement

The raw data supporting the conclusions of this article will be made available by the authors, without undue reservation.

## Ethics statement

The studies involving human participants were reviewed and approved by Institutional Review Board of National Development and Research Institutes, Inc. The participants provided their written informed consent to participate in this study.

## Author contributions

DF, HG, and PM-G conceptualized the general approach of this study. KQ and KR conducted initial analyses. DK, KG, HG, and PM-G revised the analytic plan. DK and KG conducted the final analyses reported here. HG wrote the current version of the manuscript, with assistance from DK, KG, and KQ particularly in the Methods and Results sections. SF provided critical feedback on an interim version of the manuscript. All authors contributed to the article and approved the submitted version.

## Funding

This study was funded by National Institutes of Health (NIH)/National Institute for Drug Abuse (NIDA) Grant # R01DA035146. In addition, during the preparation of this manuscript, various authors’ time was partially supported by NIDA Grant # R01DA041501 (HG, PM-G) or T32DA07233 (DF, KQ).

## Conflict of interest

The authors declare that the research was conducted in the absence of any commercial or financial relationships that could be construed as a potential conflict of interest.

## Publisher’s note

All claims expressed in this article are solely those of the authors and do not necessarily represent those of their affiliated organizations, or those of the publisher, the editors and the reviewers. Any product that may be evaluated in this article, or claim that may be made by its manufacturer, is not guaranteed or endorsed by the publisher.
